# Diagnostic Accuracy of Point-of-Care Tests to Diagnose Vitamin D Deficiency in Adults and Children: Systematic Review

**DOI:** 10.3390/diagnostics16081129

**Published:** 2026-04-09

**Authors:** Jacqueline Murphy, Youngjoo Kang, Philip J. Turner, Nia W. Roberts, Gail N. Hayward, Chris Bird, Thomas R. Fanshawe

**Affiliations:** 1 Nuffield Department of Primary Care Health Sciences, Radcliffe Observatory Quarter, Oxford OX2 6GG, UK; 2 Bodleian Health Care Libraries, John Radcliffe Hospital, Oxford OX3 9DU, UK; 3 Emergency Department, Birmingham Women’s and Children’s NHS Foundation Trust, Birmingham B15 2TG, UK

**Keywords:** diagnostic accuracy, point-of-care systems, point-of-care testing, technology, vitamin D deficiency

## Abstract

**Background/Objectives:** Compared to conventional test methods, point-of-care tests (POCTs) offer advantages for optimising care in patient groups at risk of vitamin D deficiency. However, their diagnostic accuracy in clinical settings has not previously been systematically assessed. We conducted a systematic review to assess the diagnostic accuracy of current point-of-care technology (POCT) for diagnosing vitamin D deficiency in adults and children. **Methods:** We searched Embase, MEDLINE and Web of Science on 3 December 2024 and also conducted forward and backward citation searching. We included studies from all patient groups and clinical settings where the index test had been conducted and processed at point of care, with a comparator of any laboratory reference standard test. We assessed risk of bias and applicability concerns for the included studies using published tools. The review was registered in advance (PROSPERO reference CRD42024618338). **Results:** After screening, five articles relating to four studies were included. These assessed five index POCTs against reference standard laboratory tests (liquid chromatography tandem mass spectrometry in three of the four included studies). The number of samples per comparison ranged from 6 to 20. There was variation in the level of agreement between POCT and laboratory reference standard tests. We also identified incomplete reporting of key study features, which prevented definitive assessment of several domains of the risk of bias and applicability tools. **Conclusions:** There is currently insufficient peer-reviewed evidence from clinical evaluations to recommend any particular POCT for vitamin D. Future studies should recruit adequate sample size and complete reporting of study design features and diagnostic accuracy measures.

## 1. Background

Vitamin D, a fat-soluble vitamin, is key to skeletal development through its role in calcium and phosphate metabolism [1]. Though vitamin D synthesis is possible through skin exposure to ultraviolet B (UVB), seasonal and geographic variation in UVB alongside lifestyle factors, such as clothing, sunscreen use, less time outdoors, and rising rates of obesity, contribute to increased risk of vitamin D deficiency and reliance on oral supplementation [2,3]. Globally, vitamin D deficiency is common but prevalence rates are highly variable, ranging from 5–18% in Oceania and the Americas to 24–49% in Europe, Asia and Africa [4]. Children and young people (CYP) with vitamin D deficiency can present symptoms such as abdominal pain and seizures [5]. The clinical sequelae, including rickets and osteomalacia, are debilitating and cardiomyopathy can be fatal in extreme cases [6].

Furthermore, vitamin D levels have increasingly been implicated in a wide range of other diseases. Evidence from randomised control trials (RCTs) suggests vitamin D supplementation is associated with reduced cancer mortality risk [7,8], lower incident rate of respiratory tract infections in children and adults [9,10,11], reduced incidence of type 1 diabetes and improved reversion to normoglycaemia [8,11], and reduced autoimmune disease [8,12]. Studies have also found an association between low levels of vitamin D and both asthma and eczema [13].

Although general screening is not recommended [2], there is a sizeable population of both CYP and adults who may benefit from testing due to increased risk of vitamin D deficiency. This includes: older adults, who have reduced biosynthesis and reduced mobility/exposure to the outdoors; people living with disabilities and/or chronic illnesses, such as diabetes or chronic kidney disease; people with high socioeconomic deprivation; and children and adults with darker skin pigmentation, obesity or lifestyle factors that significantly limit sun exposure [14,15,16,17,18].

Compared to conventional test methods, point-of-care tests (POCTs) offer advantages for optimising patient care in these at-risk groups. The portability and minimal training requirements (for example, by requiring only fingerprick blood samples) bring tests out of the hospital to where patients are, thus broadening access to healthcare services, particularly for groups where traditional phlebotomy is more challenging [19]. They offer rapid results, meaning that testing and diagnosis can take place in a single consultation which reduces time burden on the healthcare professional and enables prompt treatment initiation. They can also empower patients to manage their health proactively, integrating preventative healthcare into their daily lives [19,20].

We conducted a systematic review to assess the diagnostic accuracy of current point-of-care technology, when used in clinical settings, for diagnosing vitamin D deficiency and insufficiency in adults and children.

## 2. Methods

### 2.1. Search Strategy

We searched Embase (OvidSP) 1974-present, MEDLINE (OvidSP) 1946-present, and Science Citation Index and Conference Proceedings Citation Index—Science (Web of Science) 1900-present. Searches were conducted on 3rd December 2024. Full details of the search queries for each database are given in Supplementary Tables S1–S3. The search strategy included title, abstract, author keywords and subject headings relating to vitamin D and point-of-care testing. No date or language limits were applied. Additionally, forward and backward citation searching was conducted on the articles initially included from the first stage of the search [21].

### 2.2. Study Eligibility

Eligibility criteria related to the target population, vitamin D outcomes, index and reference standard tests, and assessment of diagnostic accuracy. We included studies from all patient groups (including studies where the patient group was not clearly described) in studies testing human samples in clinical settings. We excluded studies using non-human samples, and laboratory test (e.g., spiked) samples even if they had been modified from clinical samples. We included studies describing tests that measured blood levels of any form of vitamin D, either on a continuous scale or using any threshold (cut-off) for deficiency as defined by the study authors. We included studies that compared index point-of-care (or “near patient”) tests that did not require laboratory processing to any recognised reference standard for vitamin D measurement (as defined by the study authors, but typically a laboratory test such as liquid chromatography tandem mass spectrometry [LC-MS/MS]).

The primary outcome was the diagnostic accuracy of current point-of-care technology for diagnosing vitamin D deficiency and insufficiency in both adults and children, as reported using any numerical or graphical measure. Secondary outcomes included time to test result, cost, and subsequent clinical impact (such as prescription of supplementation), in studies also reporting the primary outcome of diagnostic accuracy. As our review question relates to assessments conducted in clinical settings, we excluded studies where the samples used for the index test were processed in a laboratory (i.e., not near patient or at point of care), even if the device was suitable for point-of-care testing and if the samples had been obtained but not processed at point of care in clinical settings.

### 2.3. Screening

Two researchers (JM, TF) independently screened studies for inclusion based first on titles and abstracts and subsequently on full texts. Conflicts for title and abstract screening were resolved by discussion between the two researchers. For full text screening, conflicts were resolved by consulting two additional researchers (PT, CB) until a consensus was reached. Screening was conducted using Covidence software. A summary of the eligibility criteria used during screening is given in Supplementary Tables S4 and S5.

### 2.4. Data Extraction

Data extraction items were adapted from the Preferred Reporting Items for Systematic review and Meta-Analysis of Diagnostic Test Accuracy Studies (PRISMA-DTA) checklist and included: study characteristics (research objectives, study design, clinical setting, population, vitamin D definition and threshold(s), index test information, clinical sample medium and sampling location, reference standard, and study funding); primary outcome (number of comparisons presented in the article, sample size, numerical and graphical diagnostic accuracy results for each comparison); secondary outcomes (time from sample to result, clinical impact, costs). A full list of data extraction items is given in Supplementary Table S7. The data extraction form allowed for capture of any diagnostic accuracy measure, as reported in each study. Where available we extracted results by subgroup (for example, by age or clinical groups of interest, such as pregnant women). We extracted numerical results relating to diagnostic accuracy and measures of agreement as reported by the authors of each study. The data extraction form was piloted for two included studies by two researchers (JM, YK), who subsequently conducted data extraction independently, after which consensus was reached through discussion.

### 2.5. Data Synthesis

We planned to use a diagnostic meta-analysis to pool study results. However, due to low numbers of identified studies and lack of coherence in the populations studied and diagnostic accuracy measures used, no formal statistical data synthesis was possible and so the results were instead summarised narratively. We report results only from diagnostic test accuracy comparisons. We only included comparisons reported within included studies that met the review eligibility criteria, in particular the fact that the index POCT was processed near patient (e.g., excluding comparisons using serum samples requiring laboratory processing).

### 2.6. Assessment of Methodological Quality

Methodological quality was assessed using a pre-defined checklist (given in Supplementary Tables S8 and S9) based on the Quality Assessment Tool for Diagnostic Accuracy Studies (QUADAS-2) [22], and included assessment of risk of bias and applicability concerns. In line with guidance for QUADAS-2 [22], concerns about risk of bias in study results were assessed based on: (1) whether patient selection methods were appropriate (such as avoiding limiting the included patients to those with clear diagnoses of vitamin D sufficiency or deficiency, or avoiding inappropriately excluding patients with higher risk of vitamin D deficiency); (2) whether the index and reference standard tests were interpreted without knowledge of the result from the comparator tests and used pre-defined thresholds for vitamin D deficiency/insufficiency; (3) whether the index and reference tests were conducted on samples obtained sufficiently close together in time that the vitamin D levels were unlikely to have changed between tests (“flow and timing”). Applicability concerns assessed whether the design of the included studies was appropriate to answer the review question, in terms of (1) whether the included patient population is a clinical subgroup who would be considered for vitamin D testing in routine clinical practice; (2) whether the index and reference standard tests were performed in a way to obtain results that are representative of those that would be obtained from vitamin D tests in routine clinical practice (“flow and timing”). Two researchers (JM, YK) independently completed the quality assessment checklists and conflicts were resolved through discussion.

### 2.7. Protocol and Registration

The protocol was registered in advance with the international prospective register of systematic reviews (PROSPERO), in line with the PRISMA-DTA guidelines [23] (reference number PROSPERO CRD42024618338, 27 November 2024 [24]).

## 3. Results

**Figure 1:**
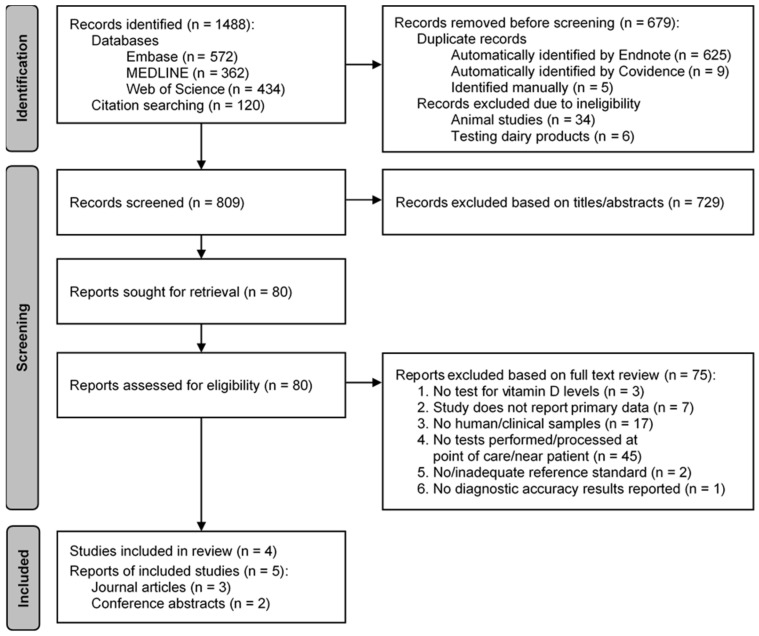
PRISMA flow diagram for selection of studies. Note: diagram modified from McInnes et al. Preferred Reporting Items for a Systematic Review and Meta-analysis of Diagnostic Test Accuracy Studies: The PRISMA-DTA Statement [23].

The database and citation searching resulted in 1488 identified studies, of which 809 were screened after removal of duplicates, and exclusion of animal-based and food testing studies. After title and abstract screening, we assessed 80 full text articles and excluded 75 of these, with the most common reason for exclusion being that tests were not performed/processed at point of care (Figure 1). Further information on reasons for exclusion of studies is given in Supplementary Table S6. There were five included articles (three journal articles and two conference abstracts) relating to four studies [25,26,27,28,29].

### 3.1. Study Characteristics

The studies were conducted in the United Kingdom (UK) [25], Malta [26,27], Portugal [28], and the United States of America (USA) [29] (Table 1).

No studies reported information on ethnicity of participants or time of year/season. The clinical setting and target population varied among included studies. One study included pregnant women attending their first appointment at an antenatal screening clinic and compared these with samples from two non-pregnant women and three quality assurance samples obtained outside the clinic [25]. Another study was conducted at a dental clinic at a university hospital, and the patient group had been selected based on pre-specified criteria relating to eligibility to receive dental implants, although these criteria were not fully described [28]. Included participants were described as 20 healthy non-smokers aged between 26 and 75 years, and patients at higher risk of dental implant failure, such as those who smoke or have diabetes, were not included [28]. For two studies the clinical setting and patient population were not described; samples were described as being derived from “convenience sampling” [26,27], from “volunteers” [29], or from “a human trial” [29].

Among the four included studies, five index POCTs were evaluated: the Vitality Health Check Quantitative Vitamin D test (Jungbrunnen—Fountain of Youth GmbH, Germany), Puredent; the RapidRead (BiotechDental; Salon-de-Provence, France); Test4D-CQ (DentaMedica; San Diego, CA, USA); Vitamin D Rapid Test Cassette (AcroBiotech Inc.); and a test based on a combination of lateral flow assay, assay reader (“TIDBIT”) and the Nutriphone smartphone app (Cornell University). The reference standard laboratory test was LC-MS/MS in all except one study, where the reference standard was described as a “standard laboratory blood test” [28]. In one study the tests were used to measure levels of vitamin D3 [29], but the form of vitamin D was unspecified in the other three studies.

Fingerprick capillary blood samples were used to evaluate the index POCT in four diagnostic accuracy comparisons from three studies [25,28,29]. In the fourth study the sampling site and sample medium for the index POCT were not reported [26,27]. Sample types for the reference standard were not well reported: intravenous blood samples in one study [26,27], blood samples from an unspecified sampling site in one study [28], and not reported in two studies [25,29]. Details of the sample processing for the reference standard (for example, whether samples were centrifuged to obtain serum before conducting the test) were poorly reported. All tests reported vitamin D as a continuous measure, and in three studies, results were also reported using thresholds to categorise vitamin D levels [25,26,27,28]. In one study diagnostic accuracy was compared at thresholds defined as: deficient <25 nmol/L, insufficient 25–50 nmol/L and sufficient >50 nmol/L [25]. In a second study, the prevalence of vitamin D deficiency was reported using a threshold of <30 ng/mL [28]. In the third study the thresholds used for classification were not defined [26,27].

### 3.2. Primary Outcome: Diagnostic Accuracy

Five diagnostic accuracy comparisons (one for each index POCT) were presented (Table 1). For all studies the unit of assessment for diagnostic accuracy was per sample; three studies stated that one sample was taken for each patient [25,26,27,28], and the fourth study did not report the number of samples per patient [29]. The total sample size among all included diagnostic accuracy comparisons was 78 samples, ranging from 6 to 20 samples per comparison.

Diagnostic accuracy results were presented using various numerical metrics and graphical formats in each of the four studies (Table 2). In one included conference abstract, Blair et al. [25] reported poor agreement (R^2^ = 0.592) between the Vitality Health Check POCT and laboratory reference standard in a mixed population of pregnant women, non-pregnant women, and external quality assurance samples (n = 12). For classification of deficiency (<25 nmol/L) in the pregnant women (n = 7), sensitivity was 100% (only one woman in the study had deficient vitamin D levels, and was correctly classified using the POCT) and specificity was 83% (5/6). For classification of insufficiency (<50 nmol/L), sensitivity was 75% (3/4) and specificity was 67% (2/3). Based on the study hospital guidelines, the authors report that five of the seven pregnant women (71%) would have been misclassified using the index POCT.

Paz et al. [28] evaluated two index POCTs (RapidRead and Test4D-CQ, n = 20 per comparison) compared to a “standard laboratory blood test”, and vitamin D levels in a cohort of healthy dental implant patients were tested before and after a six-week course of vitamin D supplementation. Using measurements from the reference standard test, 65% of the cohort were reported as vitamin D deficient at a threshold of <30 ng/mL before vitamin D supplementation [28]. Participants received both index and reference standard tests, but the results were reported using aggregate summary statistics for each test separately. The authors reported that vitamin D levels using the RapidRead POCT were on average 28.8% higher using a POCT than a laboratory reference standard before vitamin D supplementation, and 10.5% higher after supplementation. Using the Test4D-CQ POCT vitamin D levels were on average were 33.5% higher using POCT than laboratory reference standard before vitamin D supplementation, and 10.7% higher after supplementation. No paired data directly comparing the diagnostic accuracy of either index POCT to laboratory reference standard were presented.

Busuttil et al. [26,27] compared the Vitamin D Rapid Test Cassette POCT to a laboratory reference standard using samples from an undefined study population (n = 20). Concordance between tests was high (kappa = 0.84). Of two vitamin D-deficient participants, the index test correctly classified one as deficient and the other as insufficient. Index and reference test results were in agreement for the 16 vitamin D-insufficient and the 2 vitamin D-sufficient participants. For classification of deficiency, sensitivity was 50% (1/2) and specificity was 100% (18/18). For classification of insufficiency, both sensitivity and specificity were 100% (18/18 and 2/2, respectively). Definitions of the thresholds used were not reported.

The final included study [29] compared vitamin D3 levels measured by POCT (comprising a lateral flow assay, “TIDBIT” reader, and Nutriphone smartphone app) to a laboratory reference standard using blood samples from an undefined study population (n = 6). The study reported index POCT results in terms of the ratio of lateral flow “test” to “control” signal intensity (T/C ratio). Correlation between index POCT T/C ratio and laboratory reference standard vitamin D measurements was high (R^2^ = 0.94) but other diagnostic accuracy performance measures were not reported.

### 3.3. Secondary Outcomes

Three studies [26,27,28,29] reported information on time to result. For index POCTs, time to result ranged from 8 min [29] to 20 min (including consultation time) [26,27]. One study reported the time taken to conduct components of the reference laboratory test, from which the derived minimum total time was 70 min [29]. One study reported the cost of the POCT as 6 Euros per kit [26,27]. No other information on secondary outcomes was reported.

### 3.4. Methodological Quality of Included Studies

**Figure 2:**
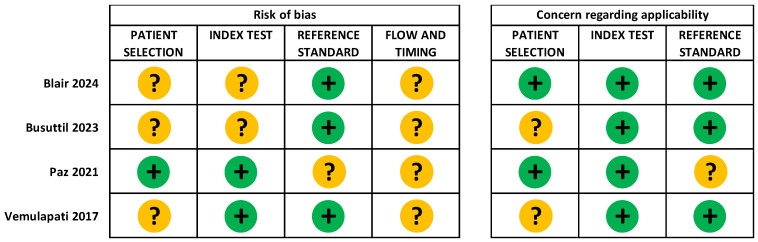
Risk of bias and concern regarding applicability assessments for included studies [25,26,27,28,29] using the QUADAS-2 framework [22]. “+” low risk of bias; “?” unclear risk of bias.

**Figure 3:**
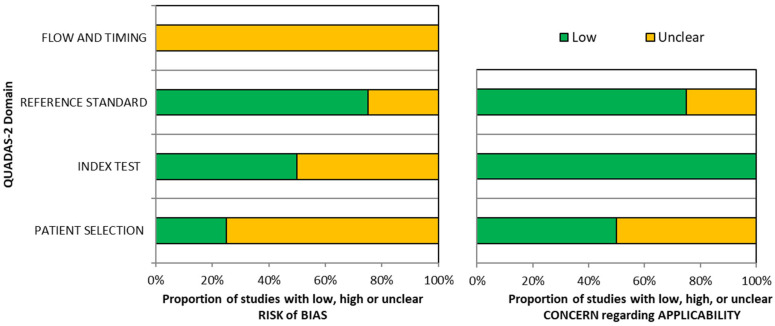
Summary of risk of bias and concern regarding applicability assessments for included studies using the QUADAS-2 framework [22].

No domains of the QUADAS-2 checklist [22] were rated as having high risk of bias or high concern that individual studies were not applicable to the review question, although many were rated as unclear because of gaps in reporting (Figure 2 and Figure 3). Regarding eligibility criteria within studies, in two studies the participants were representative of patient groups eligible for vitamin D testing in clinical practice (pregnant women [25] and systemically healthy patients awaiting dental implants [28]), so the risk of applicability for these studies was assessed as low. Of these studies, sufficient information on recruitment of participants was given for one study [28]; patients meeting standard dental implant eligibility criteria for the study clinic were consecutively recruited to the study. The clinic questionnaires to determine implant eligibility were not described, but the stated study objectives related to evaluating outcomes in routine patients at the study clinic. Therefore, based on the suitability of study recruitment procedures to address the study objectives, we assessed the risk of bias from patient selection to be low. No other assessments of methodological quality could be made relating to patient selection due to lack of reported information on study recruitment and sampling procedures [26,27,29].

The index test device and sample type were well described in all studies, and applicability to clinical practice was assessed as low concern. In three studies [25,26,27,29] the laboratory reference standard was LC-MS/MS, and was conducted as in routine clinical practice. However, in the fourth study [28] the laboratory reference standard test used was not specified. Thresholds for vitamin D deficiency/insufficiency were not defined in one of the two studies reporting classification results [26,27]; as it was not possible to determine whether the thresholds had been selected before the tests had been processed, risk of bias due to test conduct was unclear.

Timing of the index POCT and reference standard tests was generally not well described; it was unclear in most studies whether there had been a time interval or any relevant interventions between obtaining samples for each test [25,26,27,28]. Timing of tests for the fourth study [29] were reported in more detail, but it was not clear whether any recruited patients had been excluded from the study during testing. Therefore, no risk of bias assessment relating to these aspects of study design could be made for any of the included studies.

## 4. Discussion

The main finding of this review is that there is currently limited reliable evidence for the comparative diagnostic accuracy of vitamin D POCT when conducted in clinical settings at the point of care. Only four studies addressed the review question and each of these assessed a different POCT. The most common reason for exclusion of studies was that index test samples had been processed in a laboratory instead of near patient, even for studies where the index test had been developed and described as a point-of-care test. Low numbers of studies and heterogeneity in patient populations and choice of diagnostic accuracy metrics prevented statistical synthesis of the diagnostic accuracy results. In the small number of included studies there was variation in the level of agreement between POCT and laboratory reference standard tests, and also in the proportion of participants/samples misclassified as vitamin D deficient.

Characteristics of the study populations were generally not well enough described, and the number of studies too low, to present additional analysis either by clinical subgroup or taking into consideration external factors such as seasonality.

We also identified incomplete reporting of key study features, which prevented full assessment of the risk of bias and applicability of the studies for addressing the review question. In particular, information about participant recruitment, selection, and characteristics were poorly reported. In most studies we could not assess whether patient selection could have resulted in bias in diagnostic accuracy estimates, or whether the patient populations reflected groups eligible for vitamin D testing in routine clinical practice.

Several recent overviews have summarised existing tests for vitamin D, including existing and potential POCT test kit designs and performance in validation testing [30,31,32,33]. However, these were not systematic reviews and did not directly address the need for clinical evaluation in settings in which the tests would be used. A number of POCTs are currently in development for which our review found no eligible clinical evaluations, and this is typical of the wide evidence gap that can exist between development and implementation [34]. It is possible that comparative diagnostic accuracy studies exist for other POCTs which either did not meet our screening inclusion criteria or had been published in non-peer-reviewed grey literature and were therefore not captured by our search strategy. The findings of this review highlight a number of priorities for ensuring the design, conduct, and reporting of future diagnostic accuracy studies for such POCTs is adequate to contribute to the existing evidence base.

Current evidence and recommendations advise against screening the general population for vitamin D deficiency and/or insufficiency, advocating instead for routine supplementation in CYP [3], pregnant women [35], and the elderly [36]. Current clinical guidance recommends testing for children with non-specific symptoms (e.g., poor growth), infants with darker skin pigmentation who live at higher latitudes in winter and spring, CYP on either anti-convulsant or glucocorticoid therapy and CYP with malabsorption syndromes [37,38]. In adults, testing is recommended for those with suspected or known osteomalacia, osteoporosis, or symptoms suggestive of vitamin D deficiency [39].

The authors of one study in Glasgow found increasing prevalence of vitamin D deficiency with clinical sequelae presenting to the children’s hospital and theorised that a breakdown in public health measures around supplementation could underlie their findings [40]. The uncertainty between current public health guidance and frontline clinical practice may be driving the increase in demand for vitamin D testing [41,42,43] and increased costs for health systems in rich country settings [44]. POCT has the potential to rationalise testing, targeting at-risk populations to diagnose vitamin D deficiency requiring treatment at potentially less cost than costly hospital visits and laboratory time and could also serve as a quick, non-invasive test for use in epidemiological surveys, such as those used to evaluate the health outcomes of public health strategies [45].

### Limitations

Limitations of the identified studies have been discussed as part of the review results presented here. In particular, there were a number of limitations in the reporting of the included studies. For example, one study omitted information on the analytical method used for the reference standard test [28] and as a result the applicability of the diagnostic accuracy results to the review question could not be determined (as summarised in Figure 2). Our inclusion criteria allowed for any recognised reference standard, as defined by the authors of the study, and in this case we highlighted gaps in the reporting of the analytical method used for the reference standard as part of our methodological quality assessment.

Potential limitations of the review process include the strict criteria that the vitamin D tests must have been conducted and processed at point of care. This limited the final number of included studies but was consistent with our objective to evaluate POCT performance when used in a clinical setting. We identified a number of studies during screening that reported diagnostic accuracy of POCT using clinical samples from relevant patient settings, but where the POCT test had not been fully processed at the point of care. Such development studies may not produce diagnostic accuracy estimates that are truly reflective of the expected performance of the POCT in clinical practice [46], especially in studies where the blood sample is processed before testing (for example, centrifuged to obtain serum). Further studies evaluating performance in the intended clinical setting are needed in order to be able to draw conclusions about the potential level of diagnostic accuracy that POCTs might achieve in practice.

## 5. Conclusions

There is currently insufficient evidence to adequately assess the diagnostic accuracy of POCT vs. laboratory reference standard tests for diagnosing vitamin D deficiency or insufficiency in studies conducted in clinical settings at the point of care. Future studies of diagnostic accuracy in clinical settings should have adequate sample size and complete reporting of study design features, including participant characteristics, to build an evidence base for POCT that supports healthcare systems to deliver better care for this common problem.

## Figures and Tables

**Table 1:** Summary of included studies.

Summary by Study	
Number of included studies	4
Country:	
UK	1
Malta	1
Portugal	1
USA	1
Setting:	
Secondary care (antenatal clinic)	1
Secondary care (dental clinic)	1
Not reported	2
Population:	
Pregnant women	1
Patients awaiting dental implants	1
Not reported	2
Vitamin D outcome:	
Vitamin D/“25-hydroxy vitamin D (25(OH)D)”/“25-OH vitamin D”	3
Vitamin D3/25(OH)D3	1
Vitamin D threshold definitions:	
Deficient < 25 nmol/L, insufficient 25–50 nmol/L, sufficient > 50 nmol/L	1
Deficient < 30 ng/mL	1
Thresholds used but not defined	1
No thresholds used (continuous result only)	1
**Summary by diagnostic accuracy comparison**	
Total number of diagnostic accuracy comparisons	5
Sample size:	
Total across all comparisons	78
Median (IQR) [range] per comparison	20 (12,20) [6,20]
Index POCT	
Test name:	
Vitality Health Check Quantitative Vitamin D test	1
Vitamin D Rapid Test Cassette	1
RapidRead	1
Test4D-CQ	1
Lateral flow assay + “TIDBIT” reader + Nutriphone smartphone app	1
Sample site/medium:	
Fingerprick blood	4
Not reported	1
Reference standard	
Type of laboratory test:	
LC-MS/MS	3
Unspecified laboratory test	2
Sample site/medium:	
Intravenous blood	1
Blood (sample site not reported)	2
Not reported	2

IQR: interquartile range; LC-MS/MS: liquid chromatography tandem mass spectrometry; POCT: point-of-care technology; UK: United Kingdom; USA: United States of America.

**Table 2:** Summary of diagnostic accuracy comparisons in included studies.

Study	Blair 2024 [25]	Busuttil 2023 [26,27]	Paz 2021 [28]		Vemulapati 2017 [29]
Report type	Conference abstract	Conference abstract and journal article	Journal article		Journal article
Country	UK	Malta	Portugal		USA
Index POCT (sample site/medium)	Vitality Health Check (fingerprick blood)	Vitamin D Rapid Test Cassette (site/medium not reported)	RapidRead (fingerprick blood)	Test4D-CQ (fingerprick blood)	Lateral flow assay + “TIDBIT” reader + Nutriphone smartphone app (fingerprick blood)
Reference standard (sample site/medium)	LC-MS/MS (site/medium not reported)	LC-MS/MS (intravenous blood)	Laboratory test (unspecified) (blood, site not reported)	Laboratory test (unspecified) (blood, site not reported)	LC-MS/MS (site/medium not reported)
Sample size	12	20	20	20	6
Primary outcome— diagnostic accuracy (TP, FP, TN, FN) [sensitivity, specificity] or Mean (SD); median [IQR]; (min, max), in micro-g/mL	All samples (n = 12) R-squared = 0.592 Pregnant women (n = 7) Classification accuracy to detect low vitamin D levels: Threshold: deficient (<25 nmol/L) (1, 1, 5, 0) [100%, 83.3%] Threshold: insufficient (<50 nmol/L) (3, 1, 2, 1) [75%, 66.7%]	Concordance kappa = 0.84 Classification accuracy to detect low vitamin D levels: Threshold: deficient * (1, 0, 18, 1) [50%, 100%] Threshold: insufficient * (18, 0, 2, 0) [100%, 100%]	Aggregate summary statistics **: Pre-supplementation: Index POCT: 31.89 (12.58); 33.10 [12.85]; (9.00, 65.00) Reference standard: 24.76 (9.21); 25.00 [11.80]; (7.00, 42.00) Index POCT average levels were 28.8% higher than reference standard. Post-supplementation: Index POCT: 55.38 (13.27); 52.50 [15.10]; (38.00, 85.50) Reference standard: 50.11 (13.86); 50.00 [18.25]; (31.30, 83.00) Index POCT average levels were 10.5% higher than reference standard. Difference pre/post-supplementation: Index POCT: 23.49 (12.39); 21.45 [12.50]; (1.40, 52.47); *p* < 0.001 Reference standard: 25.35 (10.01); 24.00 [11.35]; (8.00, 52.00); *p* < 0.001	Aggregate summary statistics **: Pre-supplementation: Index POCT: 33.05 (12.02); 32.25 [7.25]; (11.00, 70.00) Reference standard: 24.76 (9.21); 25.00 [11.80]; (7.00, 42.00) Index POCT average levels were 33.5% higher than reference standard. Post-supplementation: Index POCT: 55.45 (14.29); 54.15 [16.45]; (35.00, 87.00) Reference standard: 50.11 (13.86); 50.00 [18.25]; (31.30, 83.00) Index POCT average levels were 10.7% higher than reference standard. Difference pre/post-supplementation: Index POCT: 22.40 (10.95); 21.40 [8.00]; (4.00, 56.00); *p* < 0.001 Reference standard: 25.35 (10.01); 24.00 [11.35]; (8.00, 52.00); *p* < 0.001	R-squared = 0.94 Linear regression equation: Index POCT = −0.033 × reference standard + 2.9925

* Thresholds not reported; ** participants received both index and reference standard tests, but the results were reported using aggregate summary statistics for each test separately. FN: false negative; FP: false positive; LC-MS/MS: liquid chromatography tandem mass spectrometry; POCT: point-of-care technology; TN: true negative; TP: true positive; UK: United Kingdom; USA: United States of America.

## Data Availability

The original contributions presented in this study are included in the article/Supplementary Materials. Further inquiries can be directed to the corresponding author.

## Supplementary Materials

The following supporting information can be downloaded at https://www.mdpi.com/article/10.3390/diagnostics16081129/s1: Table S1: MEDLINE (OvidSP) search strategy; Table S2: Embase (OvidSP) search strategy; Table S3: Web of Science search strategy; Table S4: Eligibility criteria for title and abstract screening; Table S5: Eligibility criteria for full text screening; Table S6: Reasons for exclusion at full text review; Table S7: Data extraction form designed for the review; Table S8: Methodological quality assessment checklist; Table S9: Risk of bias assessments for included studies; Table S10: PRISMA-DTA checklist; Table S11: PRISMA-DTA for abstracts checklist.

## Abbreviations

The following abbreviations are used in this manuscript:

CYP	Children and young people
IQR	Interquartile range
LC-MS/MS	Liquid chromatography tandem mass spectrometry
POCT	Point-of-care test/technology
PRISMA-DTA	Preferred Reporting Items for Systematic review and Meta-Analysis of Diagnostic Test Accuracy Studies
QUADAS	Quality Assessment Tool for Diagnostic Accuracy Studies
RCT	Randomised control trial
UK	United Kingdom
USA	United States of America
UVB	Ultraviolet B
