# Supplementary material for: Diagnostic Accuracy of Point-of-Care Tests to Diagnose Vitamin D Deficiency in Adults and Children: Systematic Review
Source: Diagnostics (Basel). 2026 Apr 9;16(8):1129. doi: 10.3390/diagnostics16081129 (PMC13115262; doi:10.3390/diagnostics16081129)
Supplement: Supplementary file 1 [file diagnostics-16-01129-s001.zip › diagnostics-4179473-supplementary.pdf]

Diagnostic Accuracy of Point-of-Care Tests to Diagnose Vitamin D Deficiency in Adults and Children: Systematic Review

Jacqueline Murphy [1], Youngjoo Kang [1], Philip J. Turner [1], Nia W. Roberts [2], Gail N. Hayward [1], Chris Bird [1,3] and Thomas R. Fanshawe [1,*]

[1] Nuffield Department of Primary Care Health Sciences, Radcliffe Observatory Quarter, Oxford OX2 6GG, UK;
[2] Bodleian Health Care Libraries, John Radcliffe Hospital, Oxford OX3 9DU, UK;
[3] Emergency Department, Birmingham Women's and Children's NHS Foundation Trust, Birmingham B15 2TG, UK
* Correspondence: thomas.fanshawe@phc.ox.ac.uk

Supplementary information

Contents

Table S1: Medline (OvidSP) search strategy 2

Table S2: Embase (OvidSP) search strategy 2

Table S3: Web of Science search strategy 3

Table S4: Eligibility criteria for title and abstract screening 4

Table S5: Eligibility criteria for full text screening 4

Table S6: Reasons for exclusion at full text review 5

Table S7: Data extraction form designed for the review 10

Table S8: Methodological quality assessment checklist 12

Table S9: Risk of bias assessments for included studies 13

Table S10: PRISMA-DTA checklist 14

Table S11: PRISMA-DTA for abstracts checklist 16

Table S1: Medline (OvidSP) search strategy

	Search query	Results
1	exp Vitamin D/	71608
2	Vitamin D Deficiency/	20877
3	("vitamin d*" or "vit d*" or "hydroxyvitamin d*" or "25hydroxyvitamin d*" or "dihydroxyvitamin d*" or (vitamin? and d3) or 25ohd3 or "25 oh d3" or ergocalciferol* or cholecalciferol* or calcifediol* or dihydroxycholecalciferol* or calcitriol or dihyrotachysterol).ti,ab,kf.	104957
4	hypovitamin* D.ti,ab,kf.	2368
5	1 or 2 or 3 or 4	116859
6	exp Point-of-Care Systems/	21768
7	((("point of care" or poc or "point of need" or rapid* or "near patient" or bedside or bed-side or chairside or chair-side) adj5 (test* or diagnos* or immunoassay? or assay? or analysis or detect* or assessment? or screen*)) or poct).ti,ab,kf.	186981
8	(finger prick* or fingerprick*).ti,ab,kf.	1909
9	(capillary adj5 (test* or sampl* or blood or analysis)).ti,ab,kf.	20227
10	(dried blood spot? or (blood spot? adj3 (test* or sampl* or analysis))).ti,ab,kf.	6828
11	6 or 7 or 8 or 9 or 10	223392
12	5 and 11	362

Medline (Ovid MEDLINE® Epub Ahead of Print, In-Process & Other Non-Indexed Citations, Ovid MEDLINE® Daily and Ovid MEDLINE®) 1946 to present

Table S2: Embase (OvidSP) search strategy

	Search query	Results
1	exp *Vitamin D/	80788
2	Vitamin D Deficiency/	41979
3	("vitamin d*" or "vit d*" or "hydroxyvitamin d*" or "25hydroxyvitamin d*" or "dihydroxyvitamin d*" or (vitamin? and d3) or 25ohd3 or "25 oh d3" or ergocalciferol* or cholecalciferol* or calcifediol* or dihydroxycholecalciferol* or calcitriol or dihyrotachysterol).ti,ab,kf.	154356
4	hypovitamin* D.ti,ab,kf.	4073
5	1 or 2 or 3 or 4	169139
6	exp "point of care testing"/	24993
7	((("point of care" or poc or "point of need" or rapid* or "near patient" or bedside or bed-side or chairside or chair side) adj5 (test* or diagnos* or immunoassay? or assay? or analysis or detect* or assessment? or screen*)) or poct).ti,ab,kf.	234179
8	(finger prick* or fingerprick*).ti,ab,kf.	2962
9	(capillary adj5 (test* or sampl* or blood or analysis)).ti,ab,kf.	27069
10	(dried blood spot? or (blood spot? adj3 (test* or sampl* or analysis))).ti,ab,kf.	10274
11	6 or 7 or 8 or 9 or 10	280746
12	5 and 11	572

Embase (OvidSP) 1974-present

Table S3: Web of Science search strategy

	Search query	Results
1	TS=("vitamin d*" OR "vit d*" OR "hydroxyvitamin d*" OR "25hydroxyvitamin d*" OR "dihydroxyvitamin d*" OR (vitamin$ AND d3) OR 25ohd3 OR "25 oh d3" OR ergocalciferol* OR cholecalciferol* OR calcifediol* OR dihydroxycholecalciferol* OR calcitriol OR dihyrotachysterol) OR TS="hypovitamin* D" Editions: WOS.SCI,WOS.ISTP	134450
2	TS=((("point of care" OR poc OR "point of need" OR rapid* OR "near patient" OR bedside OR bed-side OR chairside OR chair-side) NEAR/5 (test* OR diagnos* OR immunoassay$ OR assay$ OR analysis OR detect* OR assessment$ OR screen*)) OR poct) OR TS=("finger prick*" OR fingerprick*) OR TS=(capillary NEAR/5 (test* OR sampl* OR blood OR analysis)) OR TS=("dried blood spot$" OR ("blood spot$" NEAR/3 (test* OR sampl* OR analysis))) Editions: WOS.SCI,WOS.ISTP	304793
3	#2 AND #1 Editions: WOS.SCI,WOS.ISTP	434

Science Citation Index and Conference Proceedings Citation Index – Science (Web of Science) 1900-present

Table S4: Eligibility criteria for title and abstract screening

Include	Exclude
Population	
• Any group providing human samples (e.g. blood, urine, saliva or any other sample type)<br>• Any age	• Non-human samples<br>• Laboratory test (or 'spiked') samples
Index test	
• Index test: vitamin D tests evaluated at the point-of-care (POCT)	• Index tests that were not performed at the point-of-care/near patient, or that require a laboratory to process the sample
Reference standard	
• Comparator (reference) test: any recognised reference standard for vitamin D measurement (as defined by study authors), typically a laboratory test e.g. for 25(OH)D	• Studies with no reference test comparator
Outcome	
• Primary: diagnostic accuracy of POCT vitamin D (cholecalciferol) level, reported using any quantitative measure, either continuously or in categories (as defined by authors, e.g. normal (50+ng/ml), insufficient (26-49 ng/ml), deficient (<25 ng/ml))<br>• Secondary: Time to result [not required for inclusion]<br>• Secondary: clinical impact (e.g. prescription of vitamin supplementation/treatment, health outcomes) [not required for inclusion]	• Studies that present no diagnostic accuracy information
Study Characteristics	
• Any study design meeting all other inclusion criteria<br>• Published at any time	• Papers such as reviews and opinion pieces that do not report primary data
Other	
Note that vitamin D may alternatively be named as any of the following: hydroxyvitamin D, 25-hydroxyvitamin D, dihydroxyvitamin D, 25(OH)D3, ergocalciferol, cholecalciferol, calcifediol, dihydroxycholecalciferol, calcitriol, dihydrotachysterol, hypovitaminosis D.	

Table S5: Eligibility criteria for full text screening

Exclusion reasons
1. No test for vitamin D levels
2. Study does not report primary data
3. No human/clinical samples
4. No tests performed/processed at point-of-care/near patient
5. No/inadequate reference standard
6. Index and comparator tests performed on different participants
7. No diagnostic accuracy results reported

Table S6: Reasons for exclusion at full text review

Citation	Title	First author	Year	DOI/article details/URL	Consensus agreement (conflicts only)	Reason for exclusion at full text screening
[47]	iQuant™ Analyser: A rapid quantitative immunoassay reader	Joseph	2017	10.1109/EMBC.2017.8037668	-	1. No test for vitamin D levels
[48]	Rapid geriatric assessment	Morley	2015	10.1016/j.jamda.2015.08.004	-	
[49]	Vitamin D and in-patient falls	Sahota	2009	10.1093/ageing/afp012	-	
[50]	Response to "importance of C-3 epimer of 25-hydroxyvitamin D in dried blood spots of neonatal population"	Bhatti	2015	10.1002/ijc.29422	-	2. Study does not report primary data
[51]	Advanced applications of green materials in biosensor	Chokkareddy	2021	10.1016/b978-0-12-820484-9.00002-7	-	
[52]	25-hydroxyvitamin D testing: immunoassays versus tandem mass spectrometry	Garg	2018	10.1016/j.cll.2018.05.007	-	
[53]	Current status of vitamin D assays: are they reliable and sufficiently informative for clinical studies?	Hewavitharana	2013	10.4155/bio.13.88	-	
[54]	Smartphone based vitamin D monitoring	Mancuso	2013	10.1159/000354245	-	
[55]	The Cornell nutriphone: Enabling precision nutrition	Mehta	2016	FASEB Journal 2016;30(Meeting Abstracts):2016 https://doi.org/10.1096/fasebj.30.1_supplement.892.19 [accessed 12 December 2024]	-	
[56]	Importance of C-3 epimer of 25-hydroxyvitamin D in dried blood spots of neonatal population	Tapan	2015	10.1002/ijc.29421	-	
[57]	Two direct (nonchromatographic) assays for 25-hydroxyvitamin D	Bouillon	1984	10.1093/clinchem/30.11.1731	Samples were created in laboratory setting.	3. No human/clinical samples
[58]	A rapid assay for 25-hydroxyvitamin-D and 1,25-dihydroxyvitamin-D 24-hydroxylase	Burgos-Trinidad	1990	10.1016/0003-2697(90)90141-u	-	
[59]	An AuNPs-based electrochemical aptasensor for the detection of 25-hydroxy vitamin D3.	Cai	2024	10.1007/s44211-023-00489-0	-	
[60]	Several approaches for vitamin D determination by surface plasmon resonance and electrochemical affinity biosensors	Carlucci	2013	10.1016/j.bios.2012.07.077	-	
[61]	Metasurface-assisted lab-on-fiber optrode for highly sensitive detection of vitamin D	Cusano	2023	10.1016/j.bios.2023.115717	-	
[62]	Gel-permeation high-performance liquid chromatography as a powerful technique for rapid analysis of calcitriol (1,25-dihydroxycholecalciferol) receptors	Danan	1983	10.1042/bj2090223	-	
[63]	A rapid and reliable test for vitamin D	Jephcott	1926	10.1042/bj0201351	-	
[64]	iQPrep Kit: A milli-fluidic test kit for immunodiagnostics	Kumarasami	2020	10.1109/memea49120.2020.9137129	-	
[65]	iQuant Auto: Automated rapid test platform for immunodiagnostics	Kumarasami	2020	10.1109/EMBC44109.2020.9176134	-	
[66]	Lemon extract supported green synthesis of bimetallic CuO/Ag nanoporous materials for sensitive detection of vitamin D3	Naikoo	2023	10.1038/s41598-023-46774-w	-	

Citation	Title	First author	Year	DOI/article details/URL	Consensus agreement (conflicts only)	Reason for exclusion at full text screening
[67]	Addressing vitamin D deficiency in infants: Development of a point-of-need test for vitamin D metabolites in breastmilk	Oeum	2020	10.1111/jpc.14831	Only used bovine milk and infant formula milk.	
[68]	Lab-on-a-chip device for rapid measurement of vitamin D levels	Peter	2018	10.1007/978-1-4939-7614-0_35	-	
[69]	Portable graphene oxide modified immunosensor for the detection of vitamin d3	Sharma	2024	10.1016/j.microc.2023.109745	The ethics section states "this research did not involve any human participants".	
[70]	Determination of vitamins D by gas-liquid chromatography. II. Rapid assay for vitamin D2 in the presence of vitamin A and E	Tsukida	1972	10.5925/jnsv1954.18.165	-	
[71]	Graphene quantum dot-gold hybrid nanoparticles integrated aptasensor for ultra-sensitive detection of vitamin D3 towards point-of-care application	Wadhwa	2020	10.1016/j.apsusc.2020.146427	-	
[72]	Paper-based microfluidic device for diagnosis of osteoporosis markers	Yerrapragada	2018	10.4155/bio-2018-0136	-	
[73]	Electrochemical detection of 25-hydroxyvitamin D3 using an oligonucleotide aptasensor	Yin	2021	10.1016/j.snb.2021.129945	The samples were spiked samples derived from human serum.	
[74]	15th international congress on pediatric laboratory medicine	[Conference proceedings]	2021	10.1515/labmed-2021-0159	-	4. No tests performed/processed at point-of-care/near patient
[75]	A rapid point-of-care assay accurately measures vitamin D	Albrecht	2021	10.1007/s40618-021-01575-8	Samples for the index test were processed to obtain serum, so not conducted near-patient.	
[76]	Ferrocene tagged primary antibody generates electrochemical signal: An electrochemical immunosensing platform for the monitoring of vitamin D deficiency in clinical samples	Anusha	2023	10.1016/j.ijbiomac.2023.124269	-	
[77]	Fabrication of handmade paper sensor based on silver-cobalt doped copolymer-ionic liquid composite for monitoring of vitamin D3 level in real samples	Anusha	2021	10.1016/j.microc.2020.105789	-	
[78]	Fabrication of electrochemical immunosensor based on GCN-β-CD/Au nanocomposite for the monitoring of vitamin D deficiency.	Anusha	2022	10.1016/j.bioelechem.2021.107935	-	
[79]	A rapid assay for 25-OH-vitamin D3 without preparative chromatography	Belsey	1974	10.1210/jcem-38-6-1046	-	
[80]	A rapid chemiluminescence immunoassay for total vitamin D status assessment in fingertip blood	Chen	2020	10.7754/Clin.Lab.2020.191034	-	
[81]	Capillary blood sampling as an alternative to venipuncture in the assessment of serum 25 hydroxyvitamin D levels	Dayre McNally	2008	10.1016/j.jsbmb.2008.08.006	-	
[82]	Standardization of a rapid assay for 25-hydroxyvitamin D and normal values in the city of Sao Paulo	Diamant	1983	Arquivos Brasileiros de Endocrinologia e Metabologia 1983;27(3):129EP – 134	-	

Citation	Title	First author	Year	DOI/article details/URL	Consensus agreement (conflicts only)	Reason for exclusion at full text screening
				[obtained from journal editorial office via email 12 December 2024]		
[83]	Comparison of capillary finger stick and venous blood sampling for 34 routine chemistry analytes: potential for in hospital and remote blood sampling	Doeleman	2024	10.1515/cclm-2024-0812	-	
[84]	Analytical and clinical validation of the 25 OH vitamin D assay for the LIAISON automated analyzer	Ersfeld	2004	10.1016/j.clinbiochem.2004.06.006	-	
[85]	Comparison of validation results of the manual and automated sample preparation for 25-hydroxyvitamin D2-D3 in LC-MS/MS analysis	Evliyaoglu	2015	10.1515/tjb-2015-s108	-	
[86]	Sample-to-answer point-of-care testing platform for quantitative detection of small molecules in blood using a smartphone-and microfluidic-based nanoplasmonic biosensor	Fan	2024	10.1016/j.cej.2024.157495	The index test was performed in a laboratory using obtained clinical samples, so not conducted near-patient.	
[87]	Methodological issues in assessing plasma 25-hydroxyvitamin D concentration in newborn infants	Gallo	2014	10.1016/j.bone.2014.01.012	-	
[88]	A high-throughput platform for the rapid screening of vitamin D status by direct infusion-MS/MS	Helmeczi	2022	10.1016/j.jlr.2022.100204	-	
[89]	Application of dried blood spots to determine vitamin D status in a large nutritional study with unsupervised sampling: the Food4Me project	Hoeller	2016	10.1017/S0007114515004298	-	
[90]	Development of a rapid LC-tandem MS assay for serum 25-hydroxyvitamin D2 and 25-hydroxyvitamin D3 and comparison with Nichols Advantage, CPBA and HPLC assays on serum from patients treated with pharmacological doses of vitamin D	Jamieson	2005	J. Bone Miner. Res. 2005;20(9):S188-S188	-	
[91]	Assay of vitamins D2 and D3, and 25-hydroxyvitamins D2 and D3 in human plasma by high-performance liquid chromatography	Jones	1978	10.1093/clinchem/24.2.287	-	
[92]	Internally calibrated quantification of protein analytes in human serum by fluorescence immunoassays in disposable elastomeric microfluidic devices	Kartalov	2008	10.1002/elps.200800297	-	
[93]	25-hydroxyvitamin D levels in serum, dried serum spots and dried blood spots	Karvaly	2016	10.1515/cclm-2016-0583	-	
[94]	Bio-inspired Ag nanovilli-based sandwich-type SERS aptasensor for ultrasensitive and selective detection of 25-hydroxy vitamin D3.	Kim	2021	10.1016/j.bios.2021.113341	-	
[95]	25-hydroxyvitamin D2/D3 analysis in human plasma using LC-MS	Koerner	2011	Spectroscopy Supplements Special Issues-03-01-2011 https://www.spectroscopyonline.com/view/25-hydroxyvitamin-d2d3-analysis-	-	

Citation	Title	First author	Year	DOI/article details/URL	Consensus agreement (conflicts only)	Reason for exclusion at full text screening
				human-plasma-using-lc-ms [accessed 12 December 2024)		
[96]	A new, highly sensitive assay for 1,25-dihydroxyvitamin D not requiring high-performance liquid chromatography: application of monoclonal antibody against vitamin D receptor to radioreceptor assay	Koyama	1992	10.1016/0003-2697(92)90426-8	-	
[97]	25-hydroxyvitamin D3 on dried blood spot: Quantification method development and validation by LC-MS/MS	Le Goff	2021	10.1515/cclm-2021-5011	-	
[98]	A smartphone platform for the quantification of vitamin D levels	Lee	2014	10.1039/c3lc51375k	-	
[99]	Enzyme immunoassay for measuring 25-hydroxyvitamin D-3 in serum	Lind	1997	10.1093/clinchem/43.6.943	-	
[100]	A LC-MS method for 25-hydroxy-vitamin D3 measurements from dried blood spots for an epidemiological survey in India	Lote-Oke	2020	10.1038/s41598-020-76955-w	-	
[101]	Development of a noninvasive vitamin D screening tool	Lukaszuk	2012	10.1111/j.1552-3934.2011.02107.x	-	
[102]	Disposable impedimetric nano-immunochips for the early and rapid diagnosis of vitamin-D deficiency	Magar	2022	10.1016/j.biosx.2022.100124	-	
[103]	Comparison of vitamin D assays ability to detect 25-hydroxyvitamin D in healthy volunteers, dialysis patients, and subjects taking vitamin D2 supplements	Mullins	2018	70th AACC Annual Scientific Meeting Abstracts, 2018<br>Clinical Chemistry 2018;64(Supplement 1):S256<br>https://meeting.myadlm.org/abstracts/annual-meeting-abstract-archive [accessed 13 December 2024]	-	
[104]	A liquid chromatography/tandem mass spectrometry method for determination of 25-hydroxy vitamin D2 and 25-hydroxy vitamin D3 in dried blood spots: a potential adjunct to diabetes and cardiometabolic risk screening	Newman	2009	10.1177/193229680900300118	-	
[105]	Comparative performance assessment of novel fluorescence immunoassay POCTs for measuring circulating levels of vitamin-D	Palermiti	2024	10.3390/molecules29071636	Samples for the index test were processed to obtain plasma, so not conducted near-patient.	
[106]	ASu@MNPs-based electrochemical immunosensor for vitamin D3 serum samples analysis	Polli	2023	10.1016/j.talanta.2022.123755	-	
[107]	A microassay for 1,25-dihydroxyvitamin D not requiring high performance liquid chromatography: application to clinical studies	Reinhardt	1984	10.1210/jcem-58-1-91	-	
[108]	ImageQuant: An image-based quantitative immunoassay analyzer	Shah	2017	10.1109/MeMeA.2017.7985913	-	
[109]	Label-free metal-oxide transistor biosensors for metabolite detection in human saliva	Sharma	2024	10.1002/advs.202306038	-	
[110]	Development of an enhanced chemiluminescence total 25(OH) vitamin D assay on the VITROS ECi/ECiQ immunodiagnostic	Sullivan	2011	Clinical Chemistry 2011;57(10 SUPPL. 1):A194EP - A195	-	

Citation	Title	First author	Year	DOI/article details/URL	Consensus agreement (conflicts only)	Reason for exclusion at full text screening
	system, the 3600 immunodiagnostic system and the 5600 integrated system					
[111]	A novel universal small-molecule detection platform based on antibody-controlled Cas12a switching	Sun	2024	10.1016/j.bios.2023.115897	Samples for the index test were processed to obtain serum, so not conducted near-patient.	
[112]	Quantification of 25-hydroxyvitamin D2 and D3 in Mitra R devices with volumetric absorptive microsampling technology (VAMS R) by UHPLC-HRMS for regular vitamin D status monitoring	Tuma	2023	10.1016/j.jpba.2023.115314	-	
[113]	Analytical performance of the Abbott ARCHITECT i2000 25-OH vitamin D immunoassay and establishment of seasonal reference values	Van Der Horst	2011	Clinical Chemistry 2011;57(10 SUPPL. 1):A192	-	
[114]	Development of ZnCdSe/ZnS quantum dot-based fluorescence immunochromatographic assay for the rapid visual and quantitative detection 25-hydroxyvitamins D in human serum	Wang	2023	10.3389/fbioe.2023.1326254	The index test was performed in a laboratory using obtained clinical samples, so not conducted near-patient.	
[115]	A multiple assay for vitamin D metabolites without high-performance liquid chromatography	Wei	1994	10.1006/abio.1994.1503	-	
[116]	Evaluation of the ARCHITECT 25-hydroxy vitamin D assay and comparison with LC-MS/MS	Wyness	2012	Clinical Chemistry 2012;58(10 SUPPL. 1):A151EP - A152	-	
[117]	Correlation and consistency between two detection methods for serum 25 hydroxyvitamin D levels in human venous blood and capillary blood	Xing	2024	10.3389/fnut.2024.1291799	-	
[118]	Concordance of three point of care testing devices with clinical chemistry laboratory standard assays and patient-reported outcomes of blood sampling methods	Yonel	2022	10.1186/s12911-022-01999-z	For the index test dried blood spot samples were processed in a laboratory and results returned in 2-3 days, therefore not conducted near-patient.	
[119]	Estimation of vitamin D levels using a chairside diagnostic test kit in patients with gingivitis and periodontitis: a cross-sectional study	Koppolu	2023	10.4103/jispcd.JISPCD_50_23	-	5. No/inadequate reference standard
[120]	Patient acceptability of targeted risk-based detection of non-communicable diseases in a dental and pharmacy setting	Yonel	2020	10.1186/s12889-020-09649-7	-	
[121]	Early high-dose vitamin D3 for critically ill, vitamin D-deficient patients	Ginde	2019	10.1056/NEJMoa1911124	Diagnostic accuracy was not the aim of the study and was not assessed.	6. No diagnostic accuracy results reported

Table S7: Data extraction form designed for the review

Category/data extraction item		Response type	Item adapted from:
Article information			
	Author	Free text	NA
	Year	Numerical	NA
	Title	Free text	PRISMA-DTA 1
Study details			
	Research question:		
	Summary of study objectives	Free text	PRISMA-DTA 4
	Study design	Free text	PRISMA-DTA 18
	Clinical setting	Free text	PRISMA-DTA D1
	Setting (further details)	Free text	PRISMA-DTA D1
	Participant characteristics and patient subgroups	Free text	PRISMA-DTA 4; PRISMA-DTA 18
	Population:		
	Participants: eligibility criteria	Free text	PRISMA-DTA 6
	Sample size (recruited participants)	Numerical	PRISMA-DTA 18
	Sample size (included participants)	Numerical	PRISMA-DTA 18
	Index test 1:		
	[Repeat rows as necessary for each comparison reported in the article]		
	Index test: name	Free text	NA
	Index test: sample type (site and medium)	Free text	NA
	Reference test:		
	[Repeat rows as necessary for each comparison reported in the article]		
	Name of reference/comparator test 1	Free text	NA
	Sample type (site and medium) for reference/comparator test 1	Free text	NA
	Outcomes:		
	Summary of diagnostic test outcome as described in the study	Free text	PRISMA-DTA 18
	For studies reporting multiple comparisons, state which was the primary/main comparison as reported by the authors (if applicable)	Free text	NA
	Additional information		
	Funding source	Free text	PRISMA-DTA 18
PRIMARY OUTCOME: Comparative diagnostic accuracy (complete a separate section for each comparison e.g. different thresholds made in the study)			
	Number of comparisons reported in the study	Numerical	NA
	Comparison 1:		
	[Duplicate section as necessary for each comparison reported in the article]		
	Description (e.g. setting, subgroup, threshold, etc.)	Free text	NA
	Unit of assessment (e.g. per patient, per sample)	Free text	PRISMA-DTA 13
	Name & threshold (if applicable) of index test	Free text	NA
	Name & threshold (if applicable) of reference/comparator test	Free text	NA
	Sample size (included participants)*	Numerical	PRISMA-DTA 18
	TP*	Numerical	PRISMA-DTA 20
	FP*	Numerical	PRISMA-DTA 20
	FN*	Numerical	PRISMA-DTA 20
	TN*	Numerical	PRISMA-DTA 20
	Sensitivity [95% CI]*	Numerical	PRISMA-DTA 20

	Specificity [95% CI]*	Numerical	PRISMA-DTA 20
	PPV*	Numerical	PRISMA-DTA 20
	NPV*	Numerical	PRISMA-DTA 20
	Other numerical diagnostic accuracy results	Free text	NA
	List any graphical diagnostic accuracy results reported	Free text	NA
	Additional information (e.g. presence of indeterminate results)	Free text	NA
SECONDARY OUTCOMES**:			
	[Repeat rows as necessary for each comparison reported in the article]		
	Time from sample until test result	Free text	NA
	Clinical impact (e.g. prescription of vitamin supplementation/treatment, health outcomes)	Free text	NA
	Cost/economic outcomes (using whatever measure reported)	Free text	NA
	State any other outcomes reported	Free text	NA

* derived if not reported in article, if estimable. ** among papers reporting the primary outcome (diagnostic accuracy). Data extraction items were adapted from [23].

Table S8: Methodological quality assessment checklist

	Category/data extraction item	Response type
**DOMAIN 1: PATIENT SELECTION**		
Article information		
	Author / Year / Title	Free text
A. Risk of Bias		
	Was a consecutive or random sample of patients enrolled?	Yes/No/Unclear
	Was a case-control design avoided?	Yes/No/Unclear
	Did the study avoid inappropriate exclusions?	Yes/No/Unclear
	OVERALL RISK: Could the selection of patients have introduced bias?	Low/High/Unclear
B. Concerns regarding applicability		
	OVERALL CONCERN: Is there concern that the included patients do not match the review question?	Low/High/Unclear
**DOMAIN 2: INDEX TEST(S)**		
A. Risk of Bias		
	Were the index test results interpreted without knowledge of the results of the reference standard?	Yes/No/Unclear
	If a threshold was used, was it pre-specified?	Yes/No/Unclear
	OVERALL RISK: Could the conduct or interpretation of the index test have introduced bias?	Low/High/Unclear
B. Concerns regarding applicability		
	OVERALL CONCERN: Is there concern that the index test, its conduct, or interpretation differ from the review question?	Low/High/Unclear
**DOMAIN 3: REFERENCE STANDARD**		
A. Risk of Bias		
	Is the reference standard a recognised gold standard for vitamin D measurement?	Yes/No/Unclear
	Were the reference standard results interpreted without knowledge of the results of the index test?	Yes/No/Unclear
	OVERALL RISK: Could the reference standard, its conduct, or its interpretation have introduced bias?	Low/High/Unclear
B. Concerns regarding applicability		
	OVERALL CONCERN: Is there concern vitamin D level as defined by the reference standard does not match the review question?	Low/High/Unclear
**DOMAIN 4: FLOW AND TIMING**		
A. Risk of Bias		
	Describe any patients who did not receive the index test(s) and/or reference standard or who were excluded from the 2x2 table (refer to flow diagram, if available):	Free text
	Describe the time interval and any interventions between samples taken for the index test(s) and reference standard:	Free text
	Was there an appropriate interval between samples taken for the index test(s) and reference standard?	Yes/No/Unclear
	Were interventions avoided between taking samples for the index test(s) and reference standard?	Yes/No/Unclear
	Did all patients receive a reference standard?	Yes/No/Unclear
	Did patients receive the same reference standard?	Yes/No/Unclear
	Were all patients included in the analysis?	Yes/No/Unclear
	OVERALL RISK: Could the patient flow have introduced bias?	Low/High/Unclear

Checklist adapted from [22].

Table S9: Risk of bias assessments for included studies

	**Blair 2024**	**Busuttil 2023**	**Paz 2021**	**Vemulapati 2017**
**DOMAIN 1: PATIENT SELECTION**				
**A. Risk of Bias**				
Was a consecutive or random sample of patients enrolled?	Unclear	No	Yes	Unclear
Was a case-control design avoided?	Yes	Yes	Yes	Unclear
Did the study avoid inappropriate exclusions?	Unclear	Unclear	Yes	Unclear
OVERALL RISK: Could the selection of patients have introduced bias?	**Unclear**	**Unclear**	**Low**	**Unclear**
**B. Concerns regarding applicability**				
OVERALL CONCERN: Is there concern that the included patients do not match the review question?	**Low**	**Unclear**	**Low**	**Unclear**
**DOMAIN 2: INDEX TEST(S)**				
**A. Risk of Bias**				
Were the index test results interpreted without knowledge of the results of the reference standard?	Unclear	Yes	Yes	Yes
If a threshold was used, was it pre-specified?	Yes	Unclear	NA	NA
OVERALL RISK: Could the conduct or interpretation of the index test have introduced bias?	**Unclear**	**Unclear**	**Low**	**Low**
**B. Concerns regarding applicability**				
OVERALL CONCERN: Is there concern that the index test, its conduct, or interpretation differ from the review question?	**Low**	**Low**	**Low**	**Low**
**DOMAIN 3: REFERENCE STANDARD**				
**A. Risk of Bias**				
Is the reference standard a recognised gold standard for vitamin D measurement?	Yes	Yes	Unclear	Yes
Were the reference standard results interpreted without knowledge of the results of the index test?	Yes	Yes	Yes	Yes
OVERALL RISK: Could the reference standard, its conduct, or its interpretation have introduced bias?	**Low**	**Low**	**Unclear**	**Low**
**B. Concerns regarding applicability**				
OVERALL CONCERN: Is there concern vitamin D level as defined by the reference standard does not match the review question?	**Low**	**Low**	**Unclear**	**Low**
**DOMAIN 4: FLOW AND TIMING**				
**A. Risk of Bias**				
Was there an appropriate interval between samples taken for the index test(s) and reference standard?	Unclear	Unclear	Unclear	Yes
Were interventions avoided between taking samples for the index test(s) and reference standard?	Unclear	Unclear	Unclear	Yes
Did all patients receive a reference standard?	Yes	Yes	Yes	Yes
Did patients receive the same reference standard?	Yes	Yes	Unclear	Yes
Were all patients included in the analysis?	Unclear	Unclear	Yes	Unclear
OVERALL RISK: Could the patient flow have introduced bias?	**Unclear**	**Unclear**	**Unclear**	**Unclear**

Checklist adapted from [22].

Table S10: PRISMA-DTA checklist

Section/topic	#	PRISMA-DTA Checklist Item	Reported on page #
TITLE / ABSTRACT			
Title	1	Identify the report as a systematic review (+/- meta-analysis) of diagnostic test accuracy (DTA) studies.	1
Abstract	2	Abstract: See PRISMA-DTA for abstracts.	1
INTRODUCTION			
Rationale	3	Describe the rationale for the review in the context of what is already known.	1
Clinical role of index test	D1	State the scientific and clinical background, including the intended use and clinical role of the index test, and if applicable, the rationale for minimally acceptable test accuracy (or minimum difference in accuracy for comparative design).	1-2
Objectives	4	Provide an explicit statement of question(s) being addressed in terms of participants, index test(s), and target condition(s).	2
METHODS			
Protocol and registration	5	Indicate if a review protocol exists, if and where it can be accessed (e.g., Web address), and, if available, provide registration information including registration number.	1,4
Eligibility criteria	6	Specify study characteristics (participants, setting, index test(s), reference standard(s), target condition(s), and study design) and report characteristics (e.g., years considered, language, publication status) used as criteria for eligibility, giving rationale.	2-3, supp info
Information sources	7	Describe all information sources (e.g., databases with dates of coverage, contact with study authors to identify additional studies) in the search and date last searched.	2, supp info
Search	8	Present full search strategies for all electronic databases and other sources searched, including any limits used, such that they could be repeated.	supp info
Study selection	9	State the process for selecting studies (i.e., screening, eligibility, included in systematic review, and, if applicable, included in the meta-analysis).	2-3
Data collection process	10	Describe method of data extraction from reports (e.g., piloted forms, independently, in duplicate) and any processes for obtaining and confirming data from investigators.	3-4
Definitions for data extraction	11	Provide definitions used in data extraction and classifications of target condition(s), index test(s), reference standard(s) and other characteristics (e.g. study design, clinical setting).	3-4
Risk of bias and applicability	12	Describe methods used for assessing risk of bias in individual studies and concerns regarding the applicability to the review question.	4
Diagnostic accuracy measures	13	State the principal diagnostic accuracy measure(s) reported (e.g. sensitivity, specificity) and state the unit of assessment (e.g. per-patient, per-lesion).	3-4

Section/topic	#	PRISMA-DTA Checklist Item	Reported on page #
Synthesis of results	14	Describe methods of handling data, combining results of studies and describing variability between studies. This could include, but is not limited to: a) handling of multiple definitions of target condition, b) handling of multiple thresholds of test positivity, c) handling multiple index test readers, d) handling of indeterminate test results, e) grouping and comparing tests, f) handling of different reference standards	3-4
Meta-analysis	D2	Report the statistical methods used for meta-analyses, if performed.	NA
Additional analyses	16	Describe methods of additional analyses (e.g., sensitivity or subgroup analyses, meta-regression), if done, indicating which were pre-specified.	NA
RESULTS			
Study selection	17	Provide numbers of studies screened, assessed for eligibility, included in the review (and included in meta-analysis, if applicable) with reasons for exclusions at each stage, ideally with a flow diagram.	4-5
Study characteristics	18	For each included study provide citations and present key characteristics including: a) participant characteristics (presentation, prior testing), b) clinical setting, c) study design, d) target condition definition, e) index test, f) reference standard, g) sample size, h) funding sources	5-7
Risk of bias and applicability	19	Present evaluation of risk of bias and concerns regarding applicability for each study.	10-11
Results of individual studies	20	For each analysis in each study (e.g. unique combination of index test, reference standard, and positivity threshold) report 2x2 data (TP, FP, FN, TN) with estimates of diagnostic accuracy and confidence intervals, ideally with a forest or receiver operator characteristic (ROC) plot.	7-9
Synthesis of results	21	Describe test accuracy, including variability; if meta-analysis was done, include results and confidence intervals.	NA
Additional analysis	23	Give results of additional analyses, if done (e.g., sensitivity or subgroup analyses, meta-regression; analysis of index test: failure rates, proportion of inconclusive results, adverse events).	NA
DISCUSSION			
Summary of evidence	24	Summarize the main findings including the strength of evidence.	11-12
Limitations	25	Discuss limitations from included studies (e.g. risk of bias and concerns regarding applicability) and from the review process (e.g. incomplete retrieval of identified research).	12
Conclusions	26	Provide a general interpretation of the results in the context of other evidence. Discuss implications for future research and clinical practice (e.g. the intended use and clinical role of the index test).	13
FUNDING			
Funding	27	For the systematic review, describe the sources of funding and other support and the role of the funders.	13

Checklist adapted from [23].

Table S11: PRISMA-DTA for abstracts checklist

Section/topic	#	PRISMA-DTA for abstracts checklist item	Reported on page #
TITLE and PURPOSE			
Title	1	Identify the report as a systematic review (+/- meta-analysis) of diagnostic test accuracy (DTA) studies.	1
Objectives	2	Indicate the research question, including components such as participants, index test, and target conditions.	1
METHODS			
Eligibility criteria	3	Include study characteristics used as criteria for eligibility.	1
Information sources	4	List the key databases searched and the search dates.	1
Risk of bias & applicability	5	Indicate the methods of assessing risk of bias and applicability.	1
Synthesis of results	A1	Indicate the methods for the data synthesis.	NA
RESULTS			
Included studies	6	Indicate the number and type of included studies and the participants and relevant characteristics of the studies (including the reference standard).	1
Synthesis of results	7	Include the results for the analysis of diagnostic accuracy, preferably indicating the number of studies and participants. Describe test accuracy including variability; if meta-analysis was done, include summary results and confidence intervals.	NA
DISCUSSION			
Strengths and limitations	9	Provide a brief summary of the strengths and limitations of the evidence	NA see main text
Interpretation	10	Provide a general interpretation of the results and the important implications.	1
OTHER			
Funding	11	Indicate the primary source of funding for the review.	NA see main text
Registration	12	Provide the registration number and the registry name	1

Checklist adapted from [23].

References

47. Joseph J, Vasan JK, Shah M, Sivaprakasam M and Mahajan L. iQuant Analyser: A rapid quantitative immunoassay reader. Annu Int Conf IEEE Eng Med Biol Soc. 2017;2017:3732-6.
48. Morley JE and Adams EV. Rapid Geriatric Assessment. J. Am. Med. Dir. Assoc. 2015;16:808-12.
49. Sahota O. Vitamin D and in-patient falls. Age Ageing. 2009;38:339-40.
50. Bhatti P, Doody DR, McKean-Cowdin R and Mueller BA. Response to "importance of C-3 epimer of 25-hydroxyvitamin D in dried blood spots of neonatal population". Int J Cancer. 2015;137:751.
51. Chokkareddy R, Kanchi S, Thakur S and Hussein FH. Advanced applications of green materials in biosensor. In: Ahmed S, editor. Applications of Advanced Green Materials: Woodhead Publishing in Materials; 2021. p. 33-75.
52. Garg U. 25-Hydroxyvitamin D Testing: Immunoassays Versus Tandem Mass Spectrometry. Clin Lab Med. 2018;38:439-53.
53. Hewavitharana AK. Current status of vitamin D assays: are they reliable and sufficiently informative for clinical studies? Bioanalysis. 2013;5:1325-7.
54. Abstracts of the 20th International Congress of Nutrition. Granada, Spain. September 15-20, 2013. Ann Nutr Metab. 2013;63 Suppl 1:1-1959.
55. Mehta S and Erickson DC. The Cornell NutriPhone: Enabling Precision Nutrition. The FASEB Journal. 2016;30:892.19-.19.
56. Tapan S, Sertoglu E and Uyanik M. Importance of C-3 epimer of 25-hydroxyvitamin D in dried blood spots of neonatal population. Int J Cancer. 2015;137:750.
57. Bouillon R, Vanherck E, Jans I, Tan BK, Vanbaelen H and Demoor P. 2 DIRECT (NONCHROMATOGRAPHIC) ASSAYS FOR 25-HYDROXYVITAMIN-D. Clin. Chem. 1984;30:1731-6.
58. Burgos-Trinidad M, Brown AJ and DeLuca HF. A rapid assay for 25-hydroxyvitamin D and 1,25-dihydroxyvitamin D 24-hydroxylase. Anal Biochem. 1990;190:102-7.
59. Cai T, Chen M, Yang J, Tang C, Lu X, Wei Z, Jiang H, Hou Y, Zhao J and Yu P. An AuNPs-based electrochemical aptasensor for the detection of 25-hydroxy vitamin D(3). Anal Sci. 2024;40:599-607.
60. Carlucci L, Favero G, Tortolini C, Di Fusco M, Romagnoli E, Minisola S and Mazzei F. Several approaches for vitamin D determination by surface plasmon resonance and electrochemical affinity biosensors. Biosens Bioelectron. 2013;40:350-5.
61. Cusano AM, Quero G, Vaiano P, Cicatiello P, Principe M, Micco A, Ruvo M, Consales M and Cusano A. Metasurface-assisted Lab-on-fiber optrode for highly sensitive detection of vitamin D. Biosens Bioelectron. 2023;242:115717.
62. Danan JL and Mathieu H. Gel-permeation high-performance liquid chromatography as a powerful technique for rapid analysis of calcitriol (1,25-dihydroxycholecalciferol) receptors. Biochem J. 1983;209:223-7.
63. Jephcott H and Bacharach AL. A Rapid and Reliable Test for Vitamin D. Biochem J. 1926;20:1351-5.
64. Kumarasami R, Joseph J, Vasan JK, Pandidurai S, Sithambaram P, Sivaprakasam M and Ieee. iQPrep Kit: A milli-fluidic test kit for immunodiagnostics. Bari, ITALY: Ieee; 2020.
65. Kumarasami R, Vasan JK, Joseph J, Sithambaram P, Pandidurai S and Sivaprakasam M. iQuant Auto: Automated Rapid Test Platform for Immunodiagnostics. Annu Int Conf IEEE Eng Med Biol Soc. 2020;2020:6131-4.
66. Naikoo GA, Almashali FM, Habis FAS, Bano M, Rather JA, Hassan IU, Sheikh RA, Kannan P, Alfagih IM and Tambuwala MM. Lemon extract supported green synthesis of bimetallic CuO/Ag nanoporous materials for sensitive detection of vitamin D3. Sci Rep. 2023;13:20482.
67. Oeum M, Hughes WJ, Wark JD, Anderson JM, Licciardi PV, Greaves R, Mulholland EK and Rajapaksa AE. Addressing vitamin d deficiency in infants: Development of a point-of-need test for vitamin d metabolites in breastmilk. Journal of Paediatrics and Child Health. 2020;56:35.
68. Peter H, Bistolas N, Schumacher S, Laurisch C, Guest PC, Holler U and Bier FF. Lab-on-a-Chip Device for Rapid Measurement of Vitamin D Levels. Methods Mol Biol. 2018;1735:477-86.

69. Sharma S, Gupta S, Saini AK, Chakrabarti S, Ram S, Kumar Gupta R, Shukla K, Saini RV and Kaushal A. Portable graphene oxide modified immunosensor for the detection of Vitamin D3. Microchemical Journal. 2024;197:109745.
70. Tsukida K and Saiki K. Determination of vitamins D by gas-liquid chromatography. II. Rapid assay for vitamin D2 in the presence of vitamin A and E. J Vitaminol (Kyoto). 1972;18:165-71.
71. Wadhwa S, John AT, Nagabooshanam S, Mathur A and Narang J. Graphene quantum dot-gold hybrid nanoparticles integrated aptasensor for ultra-sensitive detection of vitamin D3 towards point-of-care application. Applied Surface Science. 2020;521:146427.
72. R Yerrapragada M and Narayanan Unni H. Paper-Based Microfluidic Device for Diagnosis of Osteoporosis Markers. Bioanalysis. 2018;10:1639-49.
73. Yin S, Li Y, Hossain MN, Sun C and Kraatz H-B. Electrochemical detection of 25-hydroxyvitamin D3 using an oligonucleotide aptasensor. Sensors and Actuators B: Chemical. 2021;340:129945.
74. 15th International Congress on Pediatric Laboratory Medicine. LaboratoriumsMedizin. 2021;45.
75. Albrecht K, Lotz J, Frommer L, Lackner KJ and Kahaly GJ. A rapid point-of-care assay accurately measures vitamin D. Journal of Endocrinological Investigation. 2021;44:2485-92.
76. Anusha T, Bhavani KS, Hassan RYA and Brahman PK. Ferrocene tagged primary antibody generates electrochemical signal: An electrochemical immunosensing platform for the monitoring of vitamin D deficiency in clinical samples. International Journal of Biological Macromolecules. 2023;239:124269.
77. Anusha T, Sai Bhavani K, Shanmukha Kumar JV, Bonanni A and Brahman PK. Fabrication of handmade paper sensor based on silver-cobalt doped copolymer-ionic liquid composite for monitoring of vitamin D3 level in real samples. Microchemical Journal. 2021;161:105789.
78. Anusha T, Bhavani KS, Shanmukha Kumar JV, Brahman PK and Hassan RYA. Fabrication of electrochemical immunosensor based on GCN-β-CD/Au nanocomposite for the monitoring of vitamin D deficiency. Bioelectrochemistry. 2022;143:107935.
79. Belsey RE, DeLuca HF and Potts JT, Jr. A rapid assay for 25-OH-vitamin D3 without preparative chromatography. J Clin Endocrinol Metab. 1974;38:1046-51.
80. Chen X, Sun S, Liu Q, Ren F, Bai Z and Wang C. A Rapid Chemiluminescence Immunoassay for Total Vitamin D Status Assessment in Fingertip Blood. Clin Lab. 2020;66:01.
81. Dayre McNally J, Matheson LA, Sankaran K and Rosenberg AM. Capillary blood sampling as an alternative to venipuncture in the assessment of serum 25 hydroxyvitamin D levels. The Journal of Steroid Biochemistry and Molecular Biology. 2008;112:164-8.
82. Diamant LL, Borelli A and Cintra ABU. Standardization of a rapid assay for 25-hydroxyvitamin D and normal values in the city of Sao Paulo. Arquivos Brasileiros de Endocrinologia e Metabologia. 1983;27:129EP-34.
83. Doeleman MJH, Koster AF, Esseveld A, Kemperman H, Swart JF, de Roock S and Tiel Groenestege WM. Comparison of capillary finger stick and venous blood sampling for 34 routine chemistry analytes: potential for in hospital and remote blood sampling. Clin Chem Lab Med. 2024;18:18.
84. Ersfeld DL, Rao DS, Body JJ, Sackrison JL, Jr., Miller AB, Parikh N, Eskridge TL, Polinske A, Olson GT and MacFarlane GD. Analytical and clinical validation of the 25 OH vitamin D assay for the LIAISON automated analyzer. Clin Biochem. 2004;37:867-74.
85. Evliyaoglu O, Dumer DE and Celik M. Comparison of validation results of the manual and automated sample preparation for 25-hydroxyvitamin D2-D3 in LC-MS/MS analysis. Turkish Journal of Biochemistry. 2015;40:166.
86. Fan H, Li R, Chen Y, Da Q, Xiong C, Zhang Y, Qin Z, Liu GL and Huang L. Sample-to-answer point-of-care testing platform for quantitative detection of small molecules in blood using a smartphone-and microfluidic-based nanoplasmonic biosensor. Chemical Engineering Journal. 2024;501:157495.
87. Gallo S, Comeau K, Agellon S, Vanstone C, Sharma A, Jones G, L'Abbe M, Khamessan A, Weiler H and Rodd C. Methodological issues in assessing plasma 25-hydroxyvitamin D concentration in newborn infants. Bone. 2014;61:186-90.

88. Helmeczi E, Fries E, Perry L, Choong K, O'Hearn K, McNally D and Britz-McKibbin P. A high-throughput platform for the rapid screening of vitamin D status by direct infusion-MS/MS. Journal of Lipid Research. 2022;63:100204.
89. Hoeller U, Baur M, Roos FF, Brennan L, Daniel H, Fallaize R, Forster H, Gibney ER, Gibney M, Godlewska M, et al. Application of dried blood spots to determine vitamin D status in a large nutritional study with unsupervised sampling: the Food4Me project. Br J Nutr. 2016;115:202-11.
90. Jamieson DP, Zhang A, Mathieu J, Ray R, Kelling DG, Chen TC and Holick MF. Development of a rapid LC-tandem MS assay for serum 25-hydroxyvitamin D2 and 25-hydroxyvitamin D3 and comparison with Nichols Advantage, CPBA and HPLC assays on serum from patients treated with pharmacological doses of vitamin D. J. Bone Miner. Res. 2005;20:S188-S.
91. Jones G. Assay of vitamins D2 and D3, and 25-hydroxyvitamins D2 and D3 in human plasma by high-performance liquid chromatography. Clin Chem. 1978;24:287-98.
92. Kartalov EP, Lin DH, Lee DT, Anderson WF, Taylor CR and Scherer A. Internally calibrated quantification of protein analytes in human serum by fluorescence immunoassays in disposable elastomeric microfluidic devices. ELECTROPHORESIS. 2008;29:5010-6.
93. Karvaly GB, Molnar-Vilagos G, Patocs A, Olajos F, Kovacs K and Vasarhelyi B. 25-hydroxyvitamin D levels in serum, dried serum spots and dried blood spots. Clinical Chemistry and Laboratory Medicine. 2016;54:eA210.
94. Kim W, Park J, Kim W, Jo S, Kim M, Kim C, Park H, Bang D, Lee W and Park J. Bio-inspired Ag nanovilli-based sandwich-type SERS aptasensor for ultrasensitive and selective detection of 25-hydroxy vitamin D3. Biosensors and Bioelectronics. 2021;188:113341.
95. Koerner P and McGinley M. 25-Hydroxyvitamin D2/D3 Analysis in Human Plasma Using LC-MS. Lc Gc N. Am. 2011:8-11.
96. Koyama H, Prahl JM, Uhland A, Nanjo M, Inaba M, Nishizawa Y, Morii H, Nishii Y and DeLuca HF. A new, highly sensitive assay for 1,25-dihydroxyvitamin D not requiring high-performance liquid chromatography: application of monoclonal antibody against vitamin D receptor to radioreceptor assay. Anal Biochem. 1992;205:213-9.
97. Le Goff C, Coeme A, Peeters S and Cavalier E. 25-Hydroxyvitamin D3 on dried blood spot: Quantification method development and validation by LC-MS/MS. Clinical Chemistry and Laboratory Medicine. 2021;59:S231.
98. Lee S, Oncescu V, Mancuso M, Mehta S and Erickson D. A smartphone platform for the quantification of vitamin D levels. Lab on a Chip. 2014;14:1437-42.
99. Lind C, Chen JW and Byrjalsen I. Enzyme immunoassay for measuring 25-hydroxyvitamin D-3 in serum. Clin. Chem. 1997;43:943-9.
100. Lote-Oke R, Pawar J, Kulkarni S, Sanas P, Kajale N, Gondhalekar K, Khadilkar V, Kamat S and Khadilkar A. A LC–MS method for 25-hydroxy-vitamin D3 measurements from dried blood spots for an epidemiological survey in India. Scientific Reports. 2020;10:19873.
101. Lukaszuk JM, Prawitz AD, Johnson KN, Umoren J and Bugno TJ. Development of a Noninvasive Vitamin D Screening Tool. Family and Consumer Sciences Research Journal. 2012;40:229-40.
102. Magar HS, Brahman PK and Hassan RYA. Disposable impedimetric nano-immunochips for the early and rapid diagnosis of Vitamin-D deficiency. Biosensors and Bioelectronics: X. 2022;10:100124.
103. Mullins KE, Christenson R and Duh S. Comparison of vitamin d assays ability to detect 25-hydroxyvitamin d in healthy volunteers, dialysis patients, and subjects taking vitamin d2 supplements. Clinical Chemistry. 2018;64:S256.
104. Newman MS, Brandon TR, Groves MN, Gregory WL, Kapur S and Zava DT. A liquid chromatography/tandem mass spectrometry method for determination of 25-hydroxy vitamin D2 and 25-hydroxy vitamin D3 in dried blood spots: a potential adjunct to diabetes and cardiometabolic risk screening. J Diabetes Sci Technol. 2009;3:156-62.
105. Palermiti A, Manca A, Mastrantonio F, Maiese D, Curatolo A, Antonucci M, Simiele M, De Nicolò A and D'Avolio A. Comparative Performance Assessment of Novel Fluorescence Immunoassay POCTs for Measuring Circulating Levels of Vitamin-D. Molecules2024. p. 1636.

106. Polli F, D'Agostino C, Zumpano R, De Martino V, Favero G, Colangelo L, Minisola S and Mazzei F. ASu@MNPs-based electrochemical immunosensor for vitamin D3 serum samples analysis. Talanta. 2023;251:123755.
107. Reinhardt TA, Horst RL, Orf JW and Hollis BW. A microassay for 1,25-dihydroxyvitamin D not requiring high performance liquid chromatography: application to clinical studies. J Clin Endocrinol Metab. 1984;58:91-8.
108. Shah MI, Joseph J, Rajagopalan A and Sivaprakasam M. ImageQuant: An image-based quantitative Immunoassay Analyzer. 2017 IEEE International Symposium on Medical Measurements and Applications (MeMeA). Rochester, MN, USA: IEEE Press; 2017. p. 420–5.
109. Sharma A, Faber H, AlGhamdi WS, Naphade D, Lin Y-H, Heeney M and Anthopoulos TD. Label-Free Metal-Oxide Transistor Biosensors for Metabolite Detection in Human Saliva. Advanced Science. 2024;11:2306038.
110. Sullivan SS, Lown A, Gottermeier G, Skrobach A, Robords D, Parsells J, Foti J and Lane-Brown H. Development of an enhanced chemiluminescence total 25(OH) Vitamin D Assay on the VITROS ECi/ECiQ immunodiagnostic system, the 3600 immunodiagnostic system and the 5600 integrated system. Clinical Chemistry. 2011;57:A194EP-A5.
111. Sun T, Wang W, Wang F, Shen W, Geng L, Zhang Y, Bi M, Gong T, Liu C, Guo C, et al. A novel universal small-molecule detection platform based on antibody-controlled Cas12a switching. Biosensors and Bioelectronics. 2024;246:115897.
112. Tuma C, Thomas A, Braun H and Thevis M. Quantification of 25-hydroxyvitamin D2 and D3 in Mitra® devices with volumetric absorptive microsampling technology (VAMS®) by UHPLC-HRMS for regular vitamin D status monitoring. Journal of Pharmaceutical and Biomedical Analysis. 2023;228:115314.
113. Van Der Horst FAL, Verzijl J and Blanc I. Analytical performance of the Abbott ARCHITECT i2000 25-OH Vitamin D immunoassay and establishment of seasonal reference values. Clinical Chemistry. 2011;57:A192.
114. Wang J, Sun G, Li F, Zhu Z, Sun L, Lv P and Yue H. Development of ZnCdSe/ZnS quantum dot-based fluorescence immunochromatographic assay for the rapid visual and quantitative detection 25-hydroxyvitamins D in human serum. Frontiers in Bioengineering and Biotechnology. 2023;Volume 11 - 2023.
115. Wei S, Tanaka H, Kubo T, Ichikawa M and Seino Y. A Multiple Assay for Vitamin D Metabolites without High-Performance Liquid Chromatography. Analytical Biochemistry. 1994;222:359-65.
116. Wyness SP, Straseski JA and Roberts WL. Evaluation of the ARCHITECT 25-hydroxy vitamin D assay and comparison with LC-MS/MS. Clinical Chemistry. 2012;58:A151EP-A2.
117. Xing Y, Wang K, Ma X, Zhang H and Tian X. Correlation and consistency between two detection methods for serum 25 hydroxyvitamin D levels in human venous blood and capillary blood. Frontiers in Nutrition. 2024;Volume 11 - 2024.
118. Yonel Z, Kuningas K, Sharma P, Dutton M, Jalal Z, Cockwell P, Webber J, Narendran P, Dietrich T and Chapple ILC. Concordance of three point of care testing devices with clinical chemistry laboratory standard assays and patient-reported outcomes of blood sampling methods. BMC Medical Informatics and Decision Making. 2022;22:248.
119. Koppolu P, Alshahrani AMA, Ghawas MAY, Almuqbil MSA, Swapna LA and Almuhaydib AKH. Estimation of Vitamin D Levels Using a Chairside Diagnostic Test Kit in Patients with Gingivitis and Periodontitis: A Cross-Sectional Study. J. 2023;13:402-9.
120. Yonel Z, Yahyouche A, Jalal Z, James A, Dietrich T and Chapple ILC. Patient acceptability of targeted risk-based detection of non-communicable diseases in a dental and pharmacy setting. BMC Public Health. 2020;20:1576.
121. Ginde AA, Brower RG, Caterino JM, Finck L, Banner-Goodspeed VM, Grissom CK, Hayden D, Hough CL, Hyzy RC, Khan A, et al. Early High-Dose Vitamin D3 for Critically Ill, Vitamin D-Deficient Patients. N Engl J Med. 2019;381:2529-40.
